# Evaluation of the Use, Reasons, and Satisfaction with the Complementary Medicine among Patients Living with a Permanent Ostomy

**DOI:** 10.1155/2023/2228593

**Published:** 2023-11-21

**Authors:** Leyla Ahmadi lari, Mahlagha Dehghan, Mohammad Ali Zakeri, Parvin Mangolian shahrbabaki

**Affiliations:** ^1^Department of Anesthesiology, School of Nursing, Larestan University of Medical Sciences, Larestan, Iran; ^2^Nursing Research Center, Kerman University of Medical Sciences, Kerman, Iran; ^3^Pistachio Safety Research Center, Rafsanjan University of Medical Sciences, Rafsanjan, Iran; ^4^Clinical Research Development Unit, Ali-Ibn Abi-Talib Hospital, Rafsanjan University of Medical Sciences, Rafsanjan, Iran

## Abstract

Patients living with a permanent ostomy encounter many physical, psychological, and social challenges due to the loss of function of a part of their body. Today, as the use of alternative therapies increases, some of these patients are seeking complementary medicine to relieve the symptoms and complications of their disease. Therefore, the present study aimed to determine the use of, reasons for, and satisfaction with the complementary medicine among patients living with a permanent ostomy. This descriptive cross-sectional study was conducted on 100 patients living with a permanent ostomy who were referred to ostomy clinics in southeastern Iran. The census method was used for sampling. The research tool included a complementary medicine questionnaire. SPSS-25 was used for data analysis. The results showed that in physical methods, most of the participants used herbal medicines (83%), aromatherapy (79%), vitamin supplements (76%), and diet (66%). In mental methods, most of the participants used prayer/recourse/vow (91%) and music therapy (75%) (every day to several times a year). People with university education (*p* < 0.001), higher incomes (*p* = 0.002), and history of addiction (*p* = 0.048) used more methods of complementary medicine. Fifty-three point four of the participants used herbal medicines to reduce physical complications, 46.6% used them to improve anxiety and stress induced by ostomy, and between 66 and 80% was completely satisfied with the use of various types of complementary medicine. The study results showed that the frequency of complementary medicine use among patients living with a permanent ostomy varied from a few days to several years. Considering the use of complementary medicine by these patients, educational programs, and interventions are necessary to increase the general awareness of ostomy patients about the types of complementary and alternative medicine (CAM) and the effects of these methods.

## 1. Introduction

Ostomy surgery of the bowl is an operation that can be due to a variety of reasons, such as cancer, trauma, inflammatory bowel disease, and intestinal obstruction, and it is defined as an opening in the abdomen for fecal excretion [1]. The number of these surgeries is increasing in different parts of the world. Annually, 21015 new cases with ostomy were registered in the United Kingdom (UK) in 2018 [2]. The number of patients living with an ostomy in the US has been 750,000-1 million, with approximately 100,000 ostomy surgeries per year [3]. Among 30,000 patients with permanent ostomies in Iran, 70% have colostomy, 20% have ileostomy, and 10% have urostomy, of whom about 10% have temporary and the rest have permanent ostomies [4]. According to some reports, up to 70% of the patients experience postostomy complications, including skin irritation (76%), pouch leakage (62%), offensive odor (59%), reduction in pleasurable activity (54%), and depression/anxiety (53%). These complications have high physical, mental, psychological, social, and even economic costs for patients as well as a negative impact on their quality of life [5].

Today, most patients living with an ostomy are looking for alternative and complementary methods to reduce the disease complications and improve their quality of life [6–8]. While modern medical treatments control symptoms strengthen the condition of patients with chronic diseases, complementary and alternative medicine (CAM) is a frequently used method [9, 10]. CAM, according to the definition of the World Health Organization (WHO), is the knowledge, skills, beliefs, and native experiences in different cultures that are used to maintain health, prevent, recover, or treat physical and mental conditions [11]. CAM includes more than 1800 types, many of which have been used for thousands of years. These methods are based on the medical systems of Egyptians, Chinese, Indians, Greeks, and Native Americans [12]. CAM can be divided into a variety of alternative medical systems, including traditional Chinese medicine, homeopathy, and naturopathy. Body-mind medicine includes various relaxation techniques such as yoga, meditation, and hypnosis [13].

Biologic therapies include dietary supplements and herbal medicines; physical therapy includes massage therapy, movement therapy; and energy therapy [14]. According to a study in Denmark, almost half of the patients with ostomies had a positive attitude (45.5%), about 40% of them had a neutral attitude, and 12.4% had a negative attitude towards the use of complementary medicine [15]. Keene et al.investigated the use of CAM among patients with cancer and showed that 51% of them used CAMs. Frequent reasons for use were shown to be to influence their cancer and general health and to treat complications of the cancer or therapy [16]. Lindberg et al. examined the relationship between inflammatory bowel disease and CAM in Sweden. They showed that 48.5% of the patients with inflammatory bowel diseases used massage therapy (21.3%) and herbal medicines (18%) in the last year [8]. Bahall et al. examined the use of CAM among patients with heart diseases in Tobago and showed that 56.2% of the patients used herbal medicine (85.9%) and then spiritual therapy (61.6%) [17].

In addition to suffering from the physical complications of ostomy, Iranian patients suffer from mental and psychological problems, may quit their job, have sexual problems, do not travel and exercise, and cannot conduct religious and doctrinal rituals as well as attend holy places and mosques [18]. In addition, patients were not trained about diet and had difficulty adapting to the new life style today, Iranian patients are increasingly using the CAM to reduce their complications. Traditional Iranian medicine, a type of complementary medicine, is based on four temperaments, and it uses all the elements in the nature, including plants, minerals, and animals, to balance temperaments [19]. According to a study, more than 50% of the Iranians used at least one type of complementary and traditional medicine [20–22]. According to the results of a study in Iran, about half of the people used at least one method of CAM at 1 year before the study. Prayer and herbal remedies were the most common methods have been applied. The supreme satisfaction was in yuga/meditation, traditional medicine, and hydrotherapy. The most common reasons of selection of the CAM methods were successful past experiences of CAM, no response to conventional medicine, and lower side effects of CAM [23].

The use of CAM has long been considered, and today, healthcare providers pay more attention to it as one of the therapeutic methods. Meanwhile, studies have emphasized the effective role of providing appropriate information to the community and preventing unnecessary use of CAM [22, 24]. Also, people who use a variety of CAM methods often recommend them to others [25]. On the other hand, the use of CAM is highly dependent on the culture and lifestyle of the community, which can affect patients' use of complementary medicine and satisfaction. Therefore, this study aimed to determine the use, reasons, and satisfaction with the complementary medicine among patients living with a permanent ostomy in southeastern Iran.

## 2. Materials and Methods

### 2.1. Study Type and Setting

This cross-sectional descriptive correlational study was performed on patients with ostomy, who referred to two ostomy clinics in southeastern Iran from August 2021 to late October 2021. These clinics are the main referral centers for patients with ostomy. The two centers selected for sampling were governmental, and ostomy patients were referred to these two governmental centers from doctors' offices and hospitals.

### 2.2. Study Sample

The sample size was 100 participants by using the sample size formula (power = 80%, *p*=0.05). In this study, patients with ostomy (140 people) referred to two ostomy clinics were selected for sampling. Census sampling was done from the patients who referred to the clinic, and 100 of the 140 people participated in the study. The response rate of the participants was 0.71%. Inclusion criteria were having at least 18 years of age, ability to speak Persian, visual and auditory health, and passing of at least one year from surgery. Exclusion criterion was no response to more than one-third of the questions.

### 2.3. Study Instruments

In this study, two questionnaires of demographic and background information and complementary medicine were used in order to achieve the objectives of the research.

#### 2.3.1. Demographic and Background Information Questionnaire

It includes age, sex, height, weight, level of education, economic status, type of ostomy, duration of ostomy, reason for ostomy, marital status before and after ostomy, number of children, history of addiction, and two questions about the use of CAM.

#### 2.3.2. Complementary and Alternative Medicine Questionnaire

This is a researcher-conducted questionnaire with 24 questions about the use of various types of complementary medicine (herbs, dry cupping, wet cupping, massage, diet, acupuncture, acupressure, hydrotherapy, aromatherapy, vitamin supplements, reflexology, touch therapy, homeopathy, energy therapy, and leech therapy, as well as various methods of relaxation such as yoga and meditation, prayer, hypnosis, psychotherapy, group therapy, art therapy, music therapy, and mindfulness). The questionnaire was scored on an 8-point Likert scale ranging from never to every day. In addition, the reasons for adopting complementary medicine were measured using three options: symptom reduction, anxiety reduction, and others. The minimum score was zero, while the maximum was 168, with a higher score indicating higher use of CAM. In addition, 10 items were used in this questionnaire to measure the level of satisfaction with complementary medicine on a five-point Likert scale (completely satisfied = 4, satisfied = 3, no idea = 2, dissatisfied = 1, and completely dissatisfied = 0), with the highest score being 40 and the lowest score being zero for satisfaction of CAM.

The new and related scientific books, articles, similar research studies, and websites were used to determine the content of the questionnaire [26]. According to Dehghan et al. in Iran, the content validity was used to determine the questionnaire's validity. Ten faculty members reviewed the questionnaire and confirmed the content validity index of 0.98. In addition, the test–retest method was used to determine the reliability. The questionnaire was given to 50 individuals who met the inclusion criteria, and their scores were calculated. The same individuals recompleted the questionnaire after 2 weeks. The intraclass correlation coefficient (ICC) was 0.71 [27].

### 2.4. Data Collection

Sampling began after approval of the proposal, acquisition of the code of ethics, presentation of the letter of introduction to the ostomy clinic, and coordination with the authorities. We tried to refer to the clinic when there was no disruption in the treatment course of these patients, they were in a good mental condition and also when they had enough time to answer questions. The data collection instrument in this study was a questionnaire. The researcher used interviews to complete the questionnaires.

### 2.5. Data Analysis

SPSS-25 was used to analyze data. Frequency, percentage, mean, and standard deviation were used to describe the study variables (the reason and satisfaction with using). Inferential statistics were used to determine the relationship between the variables. Independent *t*-test and analysis of variance were used to determine the relationship between the demographic information of patients and the mean of the application of various CAMs. Significance level was considered *p* < 0.05.

### 2.6. Ethics Approval and Consent to Participate

The researcher referred to the target medical centers after receiving the code of ethics (IR.KMU.REC.1398.700) from Kerman University of Medical Sciences and a letter of introduction. The researcher obtained informed consent from patients referred to the medical center for rehabilitation, and participants who were illiterate obtained their informed consent through a legally authorized representative and legal guardian. After obtaining permission from the authorities of these centers and providing the necessary explanations about the objectives and method of the study. Patients were included in the study after their doctor confirmed that they were in good health and that they were able to answer the questionnaire items, which were asked in the form of interviews and recorded by the researcher. All methods were performed in accordance with the relevant guidelines and regulations.

## 3. Results

The mean age of the participants was 54.52 ± 15.94 years. The mean ostomy duration was 3.08 ± 3.46 years. Fifty-six % of the participants were male, while 44% were female. Ninety-eight % of them had a permanent colostomy and 68% had an ostomy due to rectal or colon cancer. All patients had information on how to treat their disease with the use of complementary medicine, and 42% received information from others (Table 1). The study results showed that the majority of the participants used herbal medicines (83%), aromatherapy (79%), vitamin supplements (76%), and diet (66%) every day or several times a year. In addition, most of them used massage (46%), reflexology (33%), and hydrotherapy (18%) several times a week or several times a year. None of the participants used acupuncture, acupressure, touch therapy, or homeopathy in the last year. The majority of the participants used prayer/recourse/vow (91%) and music therapy (75%) every day or several times a year. Furthermore, no one used hypnosis and mindfulness in the last year (Table 2).

The majority of people used wet cupping, diet, vitamin supplements, leech therapy, and dry cupping to reduce the physical complications of ostomy. Moreover, 53.4% of them used herbal medicines to reduce physical complications, and 46.6% used them to improve anxiety and stress induced by ostomy, while most of the participants used massage to improve anxiety and stress induced by ostomy. None of the participants used mental methods to reduce the physical complications of ostomy. In addition, 26–100% of the participants did not consult any physician regarding the use of complementary medicines. The majority of them consulted with physicians for the use of vitamin supplements (74%) and diets (62%) (Table 3).

Patients living with a permanent ostomy were highly satisfied with the use of complementary medicine in all aspects (access to the method (80%), easy use of the method (78%), safety of the method (75%), noninterference with daily activities (66%), reduction of physical symptoms induced by ostomy (67%), reduction of psychological symptoms due to ostomy (75%), no concerns about drug interaction (67%), feeling better after using the method (80%), inexpensiveness (83%), and time saving (80%)). Therefore, 66–80% of the participants were completely satisfied with the use of various complementary medicine methods (Table 4).

People with elementary education and uneducated ones used fewer methods of complementary medicine than other people, while people with higher incomes used more methods of the complementary medicine. People with a history of addiction used fewer methods of the complementary medicine. Moreover, participants, who obtained their information from other people, used fewer methods of the complementary medicine than other participants (Table 5).

## 4. Discussion

This study aimed to evaluate the use, reasons, and satisfaction with complementary medicine among patients living with a permanent ostomy. According to the study results, the frequency of complementary medicine among patients with a permanent ostomy varies from a few days to several years. The majority of participants used herbal medicines, aromatherapy, vitamin supplements (physical methods), as well as, prayer/recourse/vows and music therapy (mental methods) every day or several times a year. Farooqui et al. showed that cancer patients used CAMs, including dietary supplements, natural products, and multivitamins [28]. However, Albabtain et al. in Saudi Arabia found that women with breast cancer highly used complementary medicines, such as spiritual therapy and herbal medicine [29]. Different uses of CAM in different studies can be attributed to the variety of treatments in different geographical areas, as well as, differences in religious and cultural beliefs.

According to the study results, the majority of participants used a variety of the complementary medicines to reduce physical complications and improve anxiety and stress induced by ostomy. In addition, Duluklu and Çelik in Turkey showed that patients with permanent colostomy used medicinal plants, such as lavender essential oil because they were simple, inexpensive, easy-to-use, and effective in eliminating the offensive odor caused by colostomy [7]. Kiwanuka et al. in Uganda reported that patients with breast cancer were more willing to use complementary medicine [30]. According to Albabtain et al. and Almousa et al. in Saudi Arabia, patients used complementary medicine because it improved their physical and mental health and immune function [29, 31]. Erku et al. in Ethiopia also found that psychological and functional well-being was a reason mentioned by patients with cancer for the use of complementary medicines [32]. Tangkiatkumjai et al. considered dissatisfaction with modern medicine, the benefits of complementary medicine and its perceived safety as the reasons for the use of complementary medicine [33]. According to Alsharif et al., cancer patients used the complementary medicines for the disease treatment and recovery [34]. Patients with a permanent ostomy seek the CAMs because their course of treatment is difficult, and they are satisfied with the benefits of complementary medicine. However, Jameson et al. reported that patients referred to rheumatology clinics in India were unaware of the effects of these methods and their costs, so they did not like to use yoga and acupuncture, which was a painful procedure, as well [35]. According to the results and review of the literature, patients with a permanent ostomy used complementary medicine to reduce the physical complications of the ostomy, which in turn affected their mental condition. Therefore, patients with a permanent ostomy are recommended to increase their knowledge and skills in using various types of complementary medicine.

According to the study results, the majority of people who used a variety of complementary medicine methods were completely satisfied with the use of complementary medicine. Furthermore, Kaur et al. showed that 99.4% of the patients were satisfied with the complementary medicine and felt that it had a positive effect on their health [36]. According to studies in Australia, Denmark, Slovenia, Spain, Switzerland, Taiwan, and the United States, more than 80% of people were satisfied with the CAM [37]. CAMs are sought by people who have an ostomy because their treatment is difficult, and they are happy to use complementary medicine because it helps them.

The study results showed that those with higher education and income used more complementary medicine methods than other people. CAMs are not covered by insurance in Iran because people, who earn more money, use CAM more [38]. Jang et al. reported that income and education were effective in increasing the use of CAMs among patients with cancer in South Korea [39]. Ghaedi et al. also emphasized the impact of socio-economic and cultural factors on the use of CAMs, which were in turn affected by many factors, such as employment and average monthly income in cancer patients [40]. Slavin et al. in Mexico found no correlation between the level of education of women with pelvic floor dysfunction and the use of complementary medicine [41]. Possible reasons may be related to differences in demographic, clinical, behavioral, and cultural characteristics.

The study results showed that individual whose source of information was the Internet and medical staff, used complementary medicine more than other participants. This finding is consistent with that of previous studies. Ebel et al. in Germany showed that physicians and nurses, print media, and the Internet were the most important sources of information for the use of complementary medicine among cancer patients [42]. The Internet was the main source of information on the use of complementary medicine in South Korea [43]. Both family and medical staff was sources of information about complementary medicine in Nigeria. However, Kang et al. in South Korea showed that patients with breast cancer used complementary medicine because they wanted to live with their husbands longer [43]. Almousa et al. in Saudi Arabia reported that family and friends were sources the of information on the use of complementary medicine [31], although information received from other sources may be appropriate, patients must receive reliable information only from eligible professional healthcare providers [44].

The study results showed that individuals, who did not have a history of addiction, used the complementary medicine more. No relevant studies were found in this regard, but based on the results of a systematic review, the effect of the complementary medicine on addiction has been unclear or negative, so further research is needed in this regard [45].

This study had several limitations. Participants became tired of the large number of questions, so the researcher tried to provide an intimate environment and collect data when patients were in good physical and mental condition. Patients living with an ostomy were inaccessible because of their limited number, so sampling was done in the medical centers affiliated with two universities of medical sciences.

## 5. Conclusion

The study results showed that the frequency of complementary medicine use among patients with a permanent ostomy varied from a few days to several years, with physical (herbal medicines and diet) and mental (prayer/recourse/vows, music therapy) methods being the most commonly used methods of complementary medicine. Patients living with an ostomy must increase their knowledge of the types and effects of CAM. On the other hand, nurses and other healthcare providers have more contact with patients, so they can inform patients of the benefits and risks of using CAM. Improving the methods of using complementary medicine can be a cost-effective care approach for patients living with an ostomy.

## Figures and Tables

**Table 1:** Description of demographic information of patients with permanent ostomy.

Variable	*N*	%
*Gender*		
Male	56	56.0
Female	44	44.0

*Marital status (before ostomy surgery)*		
Single	17	17.0
Married	79	79.0
Widow/divorced	4	4.0

*Marital status (after ostomy surgery)*		
Single	17	17.0
Married	77	77.0
Widow/divorced	6	6.0

*Education*		
Illiterate and elementary	37	37.0
Middle and high school	22	22.0
Diploma	25	25.0
University	16	16.0

*Income*		
Less than 20 million rials	12	12.0
20 to 30 million rials	43	43.0
More than 30 million rials	45	45.0

*Number of children*		
0	16	16.0
2 to 1	18	18.0
3 to 4	35	35.0
6 to 5	18	18.0
8 to 7	13	13.0

*Addiction*		
Yes	23	23.0
No	77	77.0

*Ostomy type*		
Permanent colostomy	98	98.0
Permanent ileostomy	2	2.0

*The reason for the ostomy*		
Cancer of the colon or rectum	68	68.0
Inflammatory bowel disease	13	13.0
Diuretics	16	16.0
Other	3	3.0

*Do you have information and training about the use of complementary medicine (herbal medicines, massage, hydrotherapy, etc)?*		
Yes	100	100.0
No	0	0

*What resources do you use to meet your educational needs regarding the use of complementary medicine?*		
Internet	37	37.0
Other people's experiences	42	42.0
Medical personnel	17	17.0
Other	4	4.0

**Table 2:** Frequency and percentage of complementary medicine in patients with permanent ostomy.

	Usage during the last year (frequency/percentage)							
	Never	Rarely	Several times a year	Once a month	2 times a month	Once a week	2 to 3 times a week	Every day
*Physical practices*								
Medicinal plants	12 (12)	5 (5)	4 (4)	6 (6)	5 (5)	15 (15)	27 (27)	26 (26)
Dry cupping	92 (92)	4 (4)	4 (4)	0	0	0	0	0
Wet cupping	72 (72)	11 (11)	17 (17)	0	0	0	0	0
Massage	47 (47)	7 (7)	3 (3)	16 (16)	7 (7)	15 (15)	(5) 5	0
Diet (vegetarian, raw, etc)	30 (30)	4 (4)	4 (4)	8 (8)	9 (9)	10 (10)	10 (10)	25 (25)
Acupuncture	100 (100)	0	0	0	0	0	0	0
Acupressure	100 (100)	0	0	0	0	0	0	0
Hydrotherapy	80 (80)	2 (2)	6 (6)	10 (10)	1 (1)	1 (1)	0	0
Aromatherapy	11 (11)	10 (10)	12 (12)	11 (11)	11 (11)	(29) 29	13 (13)	3 (3)
Vitamin supplements	19 (19)	5 (5)	3 (3)	28 (28)	11 (11)	21 (21)	9 (9)	4 (4)
Reflexology	66 (66)	1 (1)	7 (7)	13 (13)	(5) 5	(6) 6	(2) 2	0
Touch therapy	100 (100)	0	0	0	0	0	0	0
Homeopathy	100 (100)	0	0	0	0	0	0	0
Energy therapy	98 (98)	1 (1)	1 (1)	0	0	0	0	0
Medicinal leech	91 (91)	1 (1)	7 (7)	1 (1)	0	0	0	0

*Mental practices*								
Various relaxation methods (yoga and meditation)	76 (76)	2 (2)	12 (12)	3 (3)	7 (7)	0	0	0
Prayer/Tawassul/Nazr	4 (4)	5 (5)	7 (7)	6 (6)	3 (3)	11 (11)	5 (5)	59 (59)
Hypnotism	100 (100)	0	0	0	0	0	0	0
Psychotherapy	83 (83)	2 (2)	3 (3)	5 (5)	3 (3)	2 (2)	1 (1)	1 (1)
Group therapy	40 (40)	6 (6)	7 (7)	9 (9)	9 (9)	24 (24)	4 (4)	1 (1)
Art therapy	59 (59)	4 (4)	6 (6)	3 (3)	5 (5)	12 (12)	3 (3)	8 (8)
Music therapy	18 (18)	7 (7)	4 (4)	12 (12)	11 (11)	7 (7)	21 (21)	20 (20)
Mindfulness	99 (99)	1 (1)	0	0	0	0	0	0

**Table 3:** Frequency and percentage of the reason for using different types of complementary medicine and consulting a doctor in patients with permanent ostomy.

Method	Reason for use (*N* (%))		Consult a doctor (*N* (%))	
	Reduction of ostomy-inducedphysical complications	Improve ostomy anxiety and stress	Yes	No
*Physical practices*				
Medicinal plants	47 (53.4)	41 (46.6)	12 (12.0)	88 (88.0)
Dry cupping	7 (88.9)	1 (11.1)	1 (1.0)	99 (99.0)
Wet cupping	28 (100.0)	0	2 (2.0)	98 (98.0)
Massage	11 (21.2)	42 (78.8)	2 (2.0)	98 (98.0)
Diet (vegetarian, raw, etc)	70 (100.0)	0	62 (62.0)	38 (38.0)
Acupuncture	0	0	0	100 (100.0)
Acupressure	0	0	0	100 (100.0)
Hydrotherapy	5 (25.0)	15 (75.0)	0	99 (100.0)
Aromatherapy	1 (1.1)	88 (98.9)	1 (1.0)	99 (99.0)
Vitamin supplements	81 (100.0)	0	74 (74.0)	26 (26.0)
Reflexology	3 (8.8)	31 (91.2)	1 (1.0)	99 (99.0)
Touch therapy	0	0	0	100 (100.0)
Homeopathy	0	0	0	100 (100.0)
Energy therapy	0	2 (100.0)	0	100 (100.0)
Medicinal leech	9 (100.0)	0	0	100 (100.0)

*Mental practices*				
Various relaxation methods (yoga and meditation)	0	24 (100.0)	0	100 (100.0)
Prayer/Tawassul/Nazr	0	96 (100.0)	1 (1.0)	99 (99.0)
Hypnotism	0	0	0	100 (100.0)
Psychotherapy	0	17 (100.0)	5 (5.0)	95 (95.0)
Group therapy	0	60 (100.0)	0	100 (100.0)
Art therapy	0	41 (100.0)	0	100 (100.0)
Music therapy	0	79 (100.0)	0	100 (100.0)
Mindfulness	0	0	0	100 (100.0)

**Table 4:** Frequency and percentage of satisfaction with the use of complementary medicine in patients with permanent ostomy.

Satisfaction with complementary medicine	*N* (%)				
	Completely satisfied	Satisfied	Dissatisfied	Completely dissatisfied	No idea
Access to the method	80 (80.0)	20 (20.0)	0	0	0
Easy use of the method	78 (78.0)	22 (22.0)	0	0	0
Safety of the method	75 (75.0)	24 (24.0)	1 (1.0)	0	0
Noninterference with daily activities	66 (66.0)	33 (33.0)	0	0	1 (1.0)
Reduction of physical symptoms induced by ostomy	67 (67.0)	24 (24.0)	2 (2.0)	0	7 (7.0)
Reduction of psychological symptoms due to ostomy	75 (75.0)	22 (22.0)	0	0	3 (3.0)
No concerns about drug interaction	67 (67.0)	32 (32.0)	0	0	1 (1.0)
Feeling better after using the method	80 (80.0)	19 (19.0)	0	1 (1.0)	0
Inexpensiveness	83 (83.0)	17 (17.0)	0	0	0
Time saving	80 (80.0)	20 (20.0)	0	0	0

**Table 5:** Application of various complementary medicine methods according to demographic information of patients with permanent ostomy.

	Application of various CAM		Statistical test	*p* value
	Mean	SD		
*Gender*				
Male	32.41	11.50	*t* = −1.96	0.053
Female	37.18	12.81		

*Marital status (before ostomy surgery)*				
Single	33.29	12.79	*F* = 0.63	0.60
Married	34.77	12.21		
Widow/divorced	34.50	14.48		

*Marital status (after ostomy surgery)*				
Single	33.24	12.78	*F* = 0.37	0.78
Married	34.68	11.87		
Widow/divorced	36.00	17.54		

*Education*				
Illiterate and elementary	28.34	9.71	*F* = 6.12	0.002
Middle and high school	38.27	12.65		
Diploma	36.12	11.27		
University	40.88	13.42		

*Income*				
Less than 20 million rials	21.25	6.70	*F* = 11.95	0.001>
20 to 30 million rials	33.77	12.38		
More than 30 million rials	38.76	10.69		

*Number of children*				
0	34.00	12.86	*F* = 1.59	0.18
1 to 2	39.17	14.49		
3 to 4	35.69	11.48		
5 to 6	31.83	11.68		
7 to 8	29.23	9.64		

*Addiction*				
Yes	30.09	10.05	*t* = −2.0	0.048
No	35.83	12.61		

*The reason for the ostomy*				
Cancer of the colon or rectum	35.44	10.92	*F* = 0.95	0.42
Inflammatory bowel disease	34.00	16.66		
Diuretics	38.00	14.00		
Other	29.46	12.67		

*What resources do you use to meet your educational needs regarding the use of complementary medicine?*				
Internet	38.81	13.38	*F* = 6.75	0.001>
Other people's experiences	28.31	9.69		
Medical personnel	38.24	8.25		
Other	44.00	14.35		

SD = standard deviation, *t* = independent *t*-test, *F* = analysis of variance.

## Data Availability

The datasets used and/or analyzed during the current study are available from the corresponding author upon reasonable request.

## Abbreviations

CAM: Complementary and alternative medicines
ICC: Intraclass correlation coefficient
UK: United Kingdom
WHO: World Health Organization.
